# Notes and Notices

**Published:** 1891-12

**Authors:** 


### NOTES AND NOTICES.

DR. M. A. A. WOLFF, of Gainesville, Texas, died at the Kansas City Homœopathic Hospital, October 7th, at the age of sixty-three years. He was the son of the grand rabbi of Copenhagen. His father is still living, at the advanced age of ninety, and his last birthday was celebrated by all Europe. As soon as the necessary data are obtained a fuller account of his life will be given.

REMOVALS.—Dr. W. C. McDowell, from Sioux City to Springfield, Missouri. Dr. W. B. Farley has located at Berwyn, Chester County, Pa. Dr. E. C. D. O'Brien, from 333 East 58th Street, to 226 East 87th Street, New York City. Dr. C. O. Boyce, from Ishpeming to Marquette, Michigan. Rev. J. Stewart Smith, M. D., from Elgin, Illinois, to 1307 Holmes Street, Kansas City, Mo.

Dr. Rufus Choate, from 310 Indiana Avenue, to corner 33d and O Streets, Washington D. C.

Dr. Harriet H. Cobb has removed from 314 Broadway, Cambridgeport, to 49 North Avenue, Cambridge, Mass.

Dr. Geo. A. Taber has removed from 103 East Main Street to 11 East Grace Street, Richmond, Va.

Printers' Ink.—We wish that every merchant would become a regular reader of *Printers' Ink*, for they would become better and more liberal advertisers, since, by the new ideas and suggestions made to them by men who have spent a lifetime in the study and handling of advertising, their advertising would prove more fruitful.—*Gazette, Lawrence, Kan., April 23d.*

College of Physicians and Surgeons, 813 West Harrison Street, Chicago.—The Illinois State Board of Health has provided that a year of study with a preceptor may be accepted as one year on a four-year course. This year is usually taken preliminary to study in a medical college. The care of a student by a busy practitioner of medicine has not always been equal to the requirements of the case. Therefore this college has undertaken to co-operate with preceptors in laying out a course of reading and a course of study of accessible animals. For further particulars, and a matriculation blank, address, Dr. Bayard Holmes, Secretary, 240 Wabash Avenue, Chicago, Ill.

Fincke's Translation of the *Organon.*—It has been proposed by a number of homœopathic physicians to publish Dr. Fincke's translation of Hahnemann's *Organon.* It will contain about 272 pages; is to be printed upon the finest paper, the best carbon ink, and bound with the best muslin. This can be done for one dollar and fifty cents ($1.50) per copy, providing a sufficient number of subscribers can be obtained. To do this it will be necessary that subscribers be prompt to send in their nanes, and the number of copies they will subscribe for. By doing so, the work can be completed in a short time, and it is desirable that it should be done as soon as possible. Address, J. R. Haynes, M. D., 120 Meridian Street, Indianapolis, Ind.

Lacto-Cereal Food.—The enterprising and progressive firm of Reed & Carnrick are again in the field with a new and valued preparation called Lacto-Cereal Food, designed for invalids, dyspeptics, convalescents, the aged, and all who suffer from impaired nutrition or retrograde tissue. This food, besides being entirely palatable, contains twenty one per cent. of albuminoids, the amount required to attain and sustain the highest bodily vigor, as has been lately demonstrated by Dr. A. H. Church in his scientific experiments on English troops.

Wells on Intermittent Fever.—We regret that the amount of other material this month will compel us to defer the next installment of Intermittent fever until January.

The Ballard Binding Klip, the advertisement of which appears on advertising page 8, is a device which is needed by every physician. By its use, pamphlets that formerly lay about, and gravitated at last into the waste-basket can now be preserved. This device is used in the office of the editor of this journal, and is found to be admirable.

The Bates Numbering Machine.—On advertising page 7, we give a cut of this very remarkable machine for printing numbers in consecutive order. The editor has seen it, and can assure the profession it is everything that is claimed for it. It must prove of great value to physicians who do much writing, either for publication or only as correspondence.

The Cleveland Homœopathic Hospital College gave a banquet October 16th, to the students. One hundred and twenty guests sat down to the table. Dr. D. H. Beckwith was the orator of the evening, and gave a graphic history of the rise and progress of the college which stands as the second homœopathic college in the world. He said: "The trustees of the Cleveland Homœopathic College examined several sites suitable for a new college. The lot next to the hospital has been purchased as the most desirable one in the city, and the corner-stone for the future structure has been laid, as all of you know. The building committee would not commence the structure until the lot was paid for and enough funds raised to justify them pushing the work forward. They now thank those who have given so liberally to the good work. Twenty-seven thousand eight hundred dollars have already been subscribed and the stone donated. The committee will begin work at once, and the structure will be pushed forward to rapid completion."

Dr. Bushrod W. James, of Philadelphia, is revising his popular work on *American Climates and Resorts*, and is preparing a second edition which he hopes to have ready for issue shortly. In it he is making comparisons of the different climates, now generally resorted to, in the whole world, with a view of differentiating the same for the various kinds of invalids and tourists.

The National Homœopathic Medical College of Chicago.—The profession will be interested to learn that a new college bearing the above title and dedicated to the teaching of pure Homœopathy has recently been started.

The plan of teaching is sufficiently indicated by the following quotation from the prospectus:

"The teaching in this college will approach the ideal. At the close of each lecture the professor will give to the class a printed list of ten questions covering every important point in the lecture just delivered; the professor will also send immediately a copy of the list of questions to the President of the Faculty. At the beginning of the next lecture the class will be quizzed from the questions given at the close of the preceding lecture. The questions for the final examination will be selected from these lists of questions.

"In dispensary practice the professors will prescribe the 'single' remedy."

A staff of thirty-four professors comprises the faculty. The President of the Board of Directors is L. D. Rogers, A. M., M. D., well known as proprietor and editor of *The People's Health Journal*, of Chicago, an influential journal devoted to a popular exposition of Homœopathy as well as of hygiene and sanitary science. For information and announcements apply to Professor Wilson A. Smith, M. D., Morgan Park, Illinois.

The new college celebrated the beginning of its career by a series of Opening Exercises on Tuesday evening, Sept. 29th, the principal address being by the President. The opening of the Chicago Baptist Hospital was celebrated conjointly with that of the college.

A PRIVATE HOMŒOPATHIC INSANE ASYLUM has been started at Sandwich, Mass., by Dr. G. E. White. He is the only homœopathic physician in New England who has ever received a license for such an asylum. The Cottage System will be followed in this asylum, new cottages being built as patients increase. The matron and assistant manager of the asylum will be Miss Alice R. Cooke. The homœopathic profession are solicited to patronize the new institution.

THE CLEVELAND MEDICAL COLLEGE moved into its new building, Bolivar Street, Cleveland, Ohio, on Wednesday, September 23d. The occasion was celebrated by a public meeting with addresses and other ceremonies, after which Professor G. J. Jones, dean of the college, opened the formal lecture session at two o'clock, and at three o'clock he was followed by Professor Jewett, and the new college was at work in its new building, successful, radiant, and happy.

The building is a large one, of brick, three stories high, finished in Norway spruce and hard woods. There is an abundance of light and ventilation. The amphitheatre occupies two stories, and will comfortably seat 200 students. In addition there are etherizing rooms, waiting rooms, janitors' rooms, and a room for the faculty, all connected with electric bells. In the amphitheatre was exhibited a large assortment of valuable surgical and gynecological instruments presented by a lady friend of the college.

ORIFICIAL SURGERY.—Dr. Pratt, of Chicago, the able originator and advocate of orificial surgery, has been subjected to some sharp and able criticism of his methods by Dr. L. Mills Fowler, of Gainesville, Texas. Dr. Fowler has since received a protest for his criticism from Dr. Lippincott. Dr. Fowler has replied in an open letter, reaffirming the doctrine of Hahnemann in some well-chosen sentences. This letter appears in full in the *Southern Journal of Homœopathy* for August, page 211.

THE KANSAS CITY HOMŒOPATHIC MEDICAL SOCIETY is an excellent society for the propagation of pure Homœopathy. Its President, Dr. Edward F. Brady, is out in an eloquent appeal for increased attendance of its members.

He says: "Our libraries and our individual experience enable us to cover a large field; but how often do we find our armor weak, then we are compelled to call our brother practitioner for counsel—the weak spot is covered and we are better equipped for future combat with the enemy—disease. What arguments can any one urge against our gathering together once each month, to commingle socially and fraternally, and exchange with each other the golden facts of our experience."

THE MISSOURI INSTITUTE OF HOMŒOPATHY held its annual session at Kansas City, Mo., April 20th, 21st, 22d. After an extended discussion upon medical subjects, the following officers were elected for the ensuing year: President, A. Cuvier Jones, of Holden; First Vice-President, T. H. Hudson, of Kansas City; Second Vice-President, H. W. Westover, of St. Joseph; Secretary, W. P. Cutler, of Kansas City; Provisional Secretary, M. T. Runnels, of Kansas City; W. B. Morgan, of St. Louis, re-elected Treasurer. For Board of Censors the following gentlemen were proposed and elected: Dr. S. Thatcher, of Oregon; Dr. Gutherz, of St. Louis; and Dr. Winchell, of Rich Hill. St. Louis was chosen for the next meeting-place.

The Texas State Society has held its annual session at Fort Worth. The following officers were elected for the ensuing year: President, Dr. C. E. Edwards, of Blanco; First Vice-President, Dr. William Mercer, of Galveston; Second Vice-President, Mrs. Dr. Ellen Keller, of Fort Worth; Secretary, Dr. H. F. Fisher, of Fort Worth; Treasurer, Dr. Thatcher, the younger, of Bowie.

Dr. C. E. Fisher was elected a delegate to the American International Congress of Homœopathy and to the Institute of Homœopathy, which met in Atlantic City.

The Illinois State Homœopathic Society has elected the following officers for the ensuing year: President, C. A. Weireck, Marseilles; First Vice-President, O. B. Blackman, Dixon; Second Vice-President, A. K. Crawford, Chicago; Third Vice-President, Lucy Waite, Chicago; Secretary, W. A. Dunn, Chicago; Treasurer, A. A. Whipple, Quincy. The Board of Censors was re-elected. The Society elected the following delegates to the American Institute of Homœopathy, which meets at Atlantic City, N. J., in June: Drs. John A. Vincent, Arnulphy, Crawford, Coutant, Weierick, Lanning, and Whipple.

The Long Island College Hospital has sent out its announcement for 1891. This institution was organized for the purpose of practically uniting a Medical School and Hospital, and the regents believe they have carried out the plan to an extent unequaled by any other school in this country. The regular course of lectures lasts six months. In order to graduate, a student will be required to attend three of these courses. The regents have appointed Joshua M. Van Cott, M. D., Professor of Histology and Pathological Anatomy in place of Dr. Frank Ferguson, who has resigned. The medical class of the present year numbered two hundred and fifty, the graduating class being eighty-two. For further information apply to J. H. Raymond, M. D., Secretary of the Faculty, Long Island College Hospital, Brooklyn, N. Y.

Ohio State Homœopathic Society has held its twenty-seventh annual session and following officers were elected for the ensuing year: President, C. D. Crank, Cincinnati; First Vice-President, M. H. Parmalee, Toledo; Second Vice-President, T. C. Barnhill, Findlay; Secretary, Thomas M. Stewart, Cincinnati; Assistant Secretary, S. R. Geiser, Cincinnati; Treasurer, C. D. Ellis, Cleveland; Necrologist, D. H. Beckwith, Cleveland; Censors; Albert Claypool, Toledo, chairman; John A. Gann, Wooster; H. E. Beebe, Sidney; N. E. Wright, Berea; Mary A. Canfield, Cleveland; Stella Hunt, Cincinatti; F. C. Steingraver, Bluffton. The next annual meeting will be held in Cincinnati in May, 1892.

